# A Major Ingredient of Green Tea Rescues Mice from Lethal Sepsis Partly by Inhibiting HMGB1

**DOI:** 10.1371/journal.pone.0001153

**Published:** 2007-11-07

**Authors:** Wei Li, Mala Ashok, Jianhua Li, Huan Yang, Andrew E. Sama, Haichao Wang

**Affiliations:** 1 Department of Emergency Medicine, North Shore University Hospital-New York University School of Medicine, Manhasset, New York, United States of America; 2 The Feinstein Institute for Medical Research, Manhasset, New York, United States of America; Medical University of South Carolina, United States of America

## Abstract

**Background:**

The pathogenesis of sepsis is mediated in part by bacterial endotoxin, which stimulates macrophages/monocytes to sequentially release early (e.g., TNF, IL-1, and IFN-γ) and late (e.g., HMGB1) pro-inflammatory cytokines. Our recent discovery of HMGB1 as a late mediator of lethal sepsis has prompted investigation for development of new experimental therapeutics. We previously reported that green tea brewed from the leaves of the plant *Camellia sinensis* is effective in inhibiting endotoxin-induced HMGB1 release.

**Methods and Findings:**

Here we demonstrate that its major component, (-)-epigallocatechin-3-gallate (EGCG), but not catechin or ethyl gallate, dose-dependently abrogated HMGB1 release in macrophage/monocyte cultures, even when given 2–6 hours post LPS stimulation. Intraperitoneal administration of EGCG protected mice against lethal endotoxemia, and rescued mice from lethal sepsis even when the first dose was given 24 hours after cecal ligation and puncture. The therapeutic effects were partly attributable to: 1) attenuation of systemic accumulation of proinflammatory mediator (e.g., HMGB1) and surrogate marker (e.g., IL-6 and KC) of lethal sepsis; and 2) suppression of HMGB1-mediated inflammatory responses by preventing clustering of exogenous HMGB1 on macrophage cell surface.

**Conclusions:**

Taken together, these data suggest a novel mechanism by which the major green tea component, EGCG, protects against lethal endotoxemia and sepsis.

## Introduction

Sepsis is a systemic inflammatory response syndrome resulted from a microbial infection. As a continuum of increasing clinical severity, severe sepsis is defined as sepsis associated with one or more acute organ dysfunctions [1]. Despite recent advances in antibiotic therapy and intensive care, sepsis is still the most common cause of death in the intensive care units, claiming approximately 225,000 victims annually in the U.S. alone. The pathogenesis of sepsis is attributable, at least in part, to dys-regulated systemic inflammatory responses characterized by excessive accumulation of various proinflammatory mediators such as interleukin (IL)-1 [2], interferon (IFN)-γ [3], nitric oxide [4], [5], and macrophage migration inhibitory factor (MIF) [6].

We recently discovered that a ubiquitous protein, high mobility group box 1 (HMGB1), is released by activated macrophages/monocytes [7]–[10], and functions as a late mediator of lethal endotoxemia and sepsis [7], [11]–[13]. Circulating HMGB1 levels are elevated in a delayed fashion (after 16–32 h) in endotoxemic and septic mice [7], [11], and in patients with sepsis [7], [14], [15]. Administration of recombinant HMGB1 to mice recapitulates many clinical signs of sepsis, including fever [16], [17], derangement of intestinal barrier function [18], and tissue injury [19], [20]. In contrast, anti-HMGB1 antibodies or inhibitors (e.g., tanshinones, ethyl pyruvate, nicotine, or stearoyl lysophosphatidylcholine) significantly protect mice against LPS-induced acute tissue injury [19], [20], and lethal endotoxemia [7], [11]–[13], [21]–[23]. Notably, these anti-HMGB1 reagents are capable of rescuing mice from lethal experimental sepsis even when the first doses are given 24 h after the onset of the disease [11]–[13], [21], [23], indicating a wider window for HMGB1-targeted therapeutic strategies. Therefore, agents proven clinically safe, and yet still capable of attenuating HMGB1 release may hold potential in the prevention and treatment of inflammatory diseases.

Throughout human history, herbal medicine has formed the basis of folk remedies for various inflammatory ailments. The use of willow bark extract to reduce pain and fever was documented by a Greek physician (Hippocrates) in the 5^th^ century BC, and the subsequent discovery of salicylic acid as its pain/fever-relief active component gave rise to the first synthetic anti-inflammatory drug, aspirin, and the birth of the pharmaceutical industry. Brewed from the leaves of the plant, *Camellia sinensis*, tea has been one of the most popular beverages for almost fifty centuries. Its daily consumption (∼120 ml/person) is second only to water [24], and has been associated with many important health benefits, such as reduction of risk of oxidative stress and damage [25], atherosclerosis [25], cancer [26], and cardiovascular diseases [27]. These healing properties of green tea are attributable to its abundant polyphenolic compounds known as catechins, such as (-)-epigallocatechin-3-gallate (EGCG), (-)-epicatechin-3-gallate (EG), (-)-epigallocatechin (EGC), and (-)-epicatechin (EC). Among them, EGCG accounts for 50-80% of the total catechin, representing approximately 50 mg in a single cup (100 ml) of green tea [28]. However, it was previously unknown if green tea catechins can attenuate endotoxin-induced HMGB1 release or cytokine activities. In this study, we evaluated the capacity of tea catechins in inhibiting endotoxin-induced HMGB1 release and/or cytokine activities, and explored their therapeutic potential in animal model of sepsis.

## Methods

### Cell culture

Murine macrophage-like RAW 264.7 cells were obtained from the American Type Culture Collection (ATCC, Rockville, MD), and primary peritoneal macrophages were isolated from Balb/C mice (male, 7–8 weeks, 20–25 grams) at 2–3 days after intraperitoneal injection of 2 ml thioglycollate broth (4%) as previously described [8], [12], [23]. Murine macrophages were pre-cultured in RPMI 1640 medium (Gibco BRL, Grand Island, NY) supplemented with 10% fetal bovine serum (FBS) and 2 mmol/L glutamine. Human peripheral blood mononuclear cells (HuPBMCs) were isolated from the blood of healthy donors (Long Island Blood Bank, Melville, NY) by density gradient centrifugation through Ficoll (Ficoll-Paque PLUS, Pharmacia, Piscataway, NJ), and cultured in RPMI 1640 supplemented with 10% heat-inactivated human serum/2 mM L-glutamine as previously described [8], [12].

### LPS stimulation

Adherent macrophages or monocytes were gently washed with, and cultured in, serum-free OPTI-MEM I medium two hours before stimulation with bacterial endotoxin (lipopolysaccharide, LPS, *E. coli 0111:B4,* Sigma-Aldrich). At 16 hours after LPS stimulation, levels of TNF, nitric oxide, and HMGB1 in the culture medium were determined as previously described [8], [12].

### Chemical sources and stock solutions

Epigallocatechin gallate (EGCG, C_22_H_18_O_11_), catechin (C, C_15_H_14_O_6_), or ethyl gallate (C_9_H_10_O_5_) were obtained from the Sigma (St. Louis, MO), and 10 mM stock solutions were prepared in water.

### Animal models of endotoxemia and sepsis

This study was approved and performed in accordance with the guidelines for the care and use of laboratory animals at the Feinstein Institute for Medical Research, Manhasset, New York. Endotoxemia was induced in Balb/C mice (male, 7–8 weeks) by intraperitoneal injection of bacterial endotoxin (LPS, 15 mg/kg) as previously described [7], [12], [23]. Sepsis was induced in male Balb/C mice (7–8 weeks, 20–25 g) by cecal ligation and puncture (CLP) as previously described [12], [23]. EGCG was administered intraperitoneally into mice at indicated doses and time points, and mice were monitored for survival for up to two weeks. In parallel experiments, mice were euthanized to collect blood at 52 h (following two doses of EGCG at +24 and +48 h) after CLP, and assayed for serum levels of TNF, HMGB1, and other cytokines. In other parallel experiments, blood was collected from 3–5 normal healthy mice, or septic mice appearing dying (*i.e., in a moribund state*, *as judged by: 1) unresponsive to external stimuli; 2) inability to maintain upright position; and 3) agonal breathing*] or non-dying (*i.e., in a non-moribund state, as indicated by: 1) responsive to external stimuli; 2) ability to maintain upright position; and 3) normal breathing*] at 52 h post CLP, and serum levels of cytokines were determined.

### TNF ELISA

The levels of TNF in the culture medium or serum were determined using commercial enzyme linked immunosorbant assay (ELISA) kits (Catalog no. MTA00, R & D Systems, Minneapolis, MN) with reference to standard curves of purified recombinant TNF at various dilutions as previously described [12], [23].

### Nitric oxide assay

The levels of nitric oxide in the culture medium were determined indirectly by measuring the NO^2−^ production with a colorimetric assay based on the Griess reaction [12], [23]. NO^2−^ concentrations were determined with reference to a standard curve generated with sodium nitrite at various dilutions.

### HMGB1 Western blotting analysis

The levels of HMGB1 in the culture medium or serum were determined by Western blotting analysis as previously described [7], [8], [12], [23]. The relative band intensity was quantified by using the NIH image 1.59 software to determine HMGB1 levels with reference to standard curves generated with purified HMGB1.

### Cytokine antibody array

Murine cytokine antibody array (Cat. No. M0308003, RayBiotech Inc., Norcross, GA, USA), which detects 62 cytokines on one membrane, was used to determine the profile of cytokines in the culture medium or serum as previously described [12]. Briefly, the membranes were sequentially incubated with equal volume of cell-conditioned culture medium, or murine serum (after 1:10 dilution), primary biotin-conjugated antibodies, and horseradish peroxidase–conjugated streptavidin. After exposing to X-ray film, the relative signal intensity was determined using the NIH image 1.59 software, and expressed as % of positive controls on the same membrane.

### Cell Viability Assays

Cell viability was assessed by trypan blue exclusion assays as previously described [8]. Briefly, trypan blue was added to cell cultures at a final concentration of 0.08%. After incubation for 5 min at 25°C, cell viability was assessed by the percentage of dye-excluding cells in five 40× microscope fields.

### Expression and purification of recombinant HMGB1

The cDNA encoding for rat HMGB1 was cloned onto a pCAL-n vector, and the recombinant plasmid was transformed into *E. coli* BL21 (DE3) pLysS cells as previously described [7]. Recombinant HMGB1 containing a ∼3 kDa calmodulin-binding peptide tag (CBP-HMGB1 fusion protein, 33 kDa) was expressed in *E. coli*, and purified to remove contaminating endotoxin using polymyxin B column as previously described [7], [29], [30]. Recombinant HMGB1 preparations were tested routinely for LPS content by the chromogenic *Limulus* amebocyte lysate assay (Endochrome; Charles River), and endotoxin content was below detection limit (<500 pg endotoxin per microgram of rHMGB1). Recombinant HMGB1 was biotinylated using a Pierce EZ-Link Sulfo-NHS-LC-Biotinylation Kit (Cat. # 21430) following the manufacturer's protocol. The sulfonated NHS esters are cell membrane-impermeable, and are therefore suitable for cell-surface binding/uptake studies. Subsequently, the biotinylated protein was purified by gel filtration chromatography using Sephadex G-25 column.

### Fluorescence Immunostaining

RAW 264.7 cells were grown to subconfluence, and incubated with biotinylated HMGB1, in the absence or presence of EGCG (10 µM) for various period of time. Subsequently, cells were fixed with 2% formalin for 10 min, and permeabilized with 0.1% Triton X-100 in PBS (1 min, room temperature). After extensive washing with PBS, cells were incubated sequentially with antigen-affinity-purified rabbit anti-HMGB1 antibodies, and goat anti-rabbit secondary antibodies conjugated with green Alexa fluor 488 (Molecular Probes, Eugene, OR). To visualize exogenous HMGB1, cells were co-incubated with streptavidin-conjugated Alexa fluor 594 or Alexa fluor 488 (Molecular Probes). Images were captured using a fluorescence microscope (Carl Zeiss Microimaging).

### Streptavidin pull-down assays

Cell lysates were incubated with streptavidin agarose beads (Cat.# 15942-050, Invitrogen) for 1.5 h at 4°C on a rotating platform. After centrifugation, agarose beads were washed six times with 1×PBS, and bound proteins were eluted with Laemmili sample buffer (Cat. # 161-0737, Bio-Rad), and analyzed by SDS-PAGE and Western blotting with anti-HMGB1 antibodies.

### Statistical Analysis

Data are expressed as mean±SD of two independent experiments in triplicates (n = 2). One-way ANOVA was used for comparison among all different groups. When the ANOVA was significant, post-hoc testing of differences between groups was performed using Tukey's test. The Kaplan-Meier method was used to compare the differences in mortality rates between groups. A *P<*value less than 0.05 was considered statistically significant.

## Results

### Tea epigallocatechin gallate (EGCG) dose-dependently attenuated endotoxin-induced release of HMGB1, but not nitric oxide

**Figure 1:**
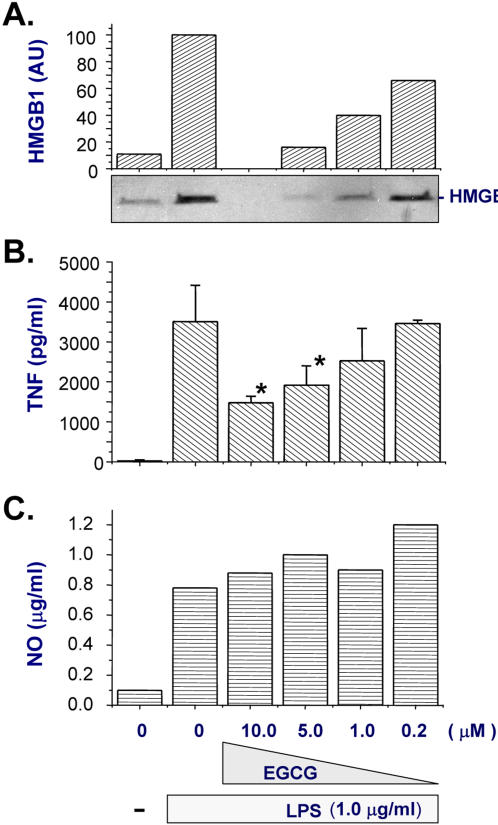
EGCG effectively abrogated endotoxin-induced HMGB1 release in murine macrophage-like RAW 264.7 cells. RAW 264.7 cells were stimulated with LPS in the absence, or presence of EGCG at indicated concentrations. At 16 hours after LPS stimulation, levels of HMGB1 (Panel A), TNF (Panel B), or nitric oxide (NO, Panel C) in the culture medium were determined by Western blotting, ELISA, and Griess reaction, respectively. Note that at concentrations that completely abrogated LPS-induced HMGB1 release (Panel A), EGCG only partially inhibited LPS-induced TNF secretion (Panel B), but preserved suppress LPS-induced nitric oxide release (Panel C).

We previously discovered that green tea brewed from the leaves of the plant, *Camellia sinensis*, is effective in inhibiting endotoxin-induced HMGB1 release [31]. To determine active components in green tea, we examined its components for HMGB1-inhibiting activities in murine macrophage-like RAW264.7 cells. A major tea catechin, EGCG, dose-dependently abrogated endotoxin-induced HMGB1 release, with an estimated IC_50_<1.0 µM (**Fig. 1A**). In contrast, at concentrations that abrogated endotoxin-induced HMGB1 release, EGCG only partially attenuated endotoxin-induced TNF secretion (**Fig. 1B**), but did not inhibit endotoxin-induced nitric oxide release (**Fig. 1C**).

**Figure 2:**
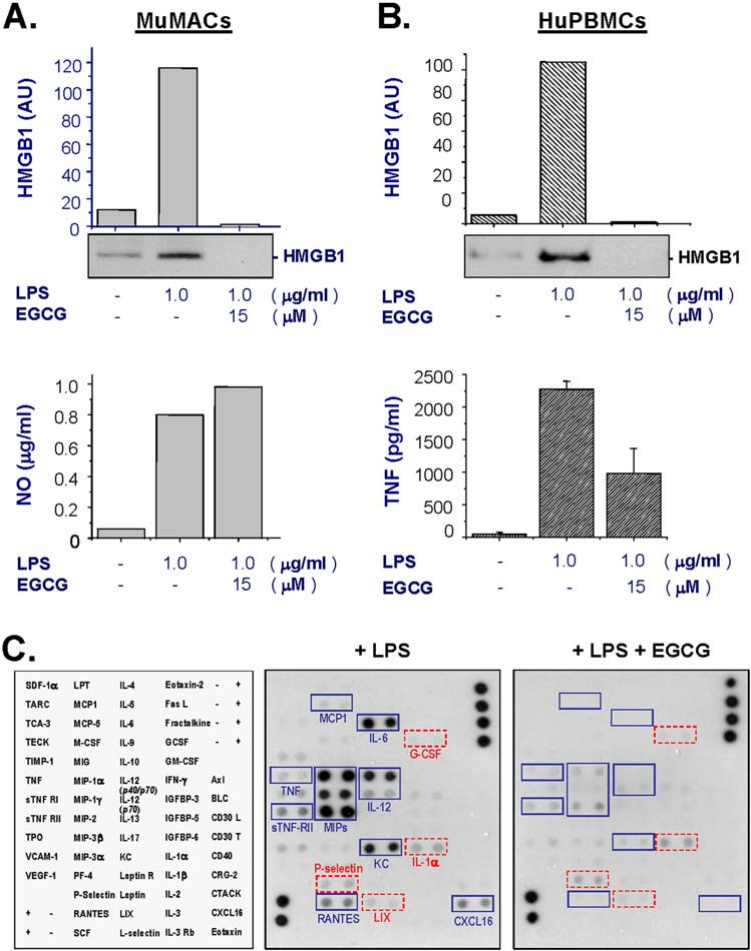
EGCG effectively abrogated endotoxin-induced HMGB1 release in primary macrophage/monocyte cultures. Primary murine peritoneal macrophages (Panel A, C), or human peripheral blood mononuclear cells (Panel B) were stimulated with LPS in the absence, or presence of EGCG (15 µM). At 16 hours after LPS stimulation, levels of HMGB1 (Panel A, B), nitric oxide (Panel A), TNF (Panel B), or other cytokines (Panel C) in the culture medium were determined by Western blotting analysis (Panel A, B, top), Griess reaction (Panel A, bottom), ELISA (Panel B, bottom), or cytokine array (Panel C), respectively. Note that at concentrations that completely abrogated LPS-induced HMGB1 release, EGCG did not block LPS-induced release of NO (Panel A), G-CSF (Panel C), IL-1α (Panel C), P-selectin (Panel C), or LIX (Panel C). In contrast, EGCG dramatically suppressed LPS-induced release of TNF, sTNF-RII, chemokines (MCP1, MIPs, KC, and RABTES), IL-6, IL-12, and CXCL16 in primary murine macrophages (Panel C). Shown in Panel C was a representative cytokine array of two independent experiments with similar results.

We further confirmed its HMGB1-inhibiting activities using primary murine peritoneal macrophages (MuMACs), as well as human peripheral blood mononuclear cells (huPBMCs). In primary MuMACs, EGCG also abrogated LPS-induced HMGB1 release, but similarly failed to inhibit LPS-induced nitric oxide release (**Fig. 2A**). In primary huPBMCs, EGCG effectively abolished LPS-induced HMGB1 release (**Fig. 2B**), and partly attenuated LPS-induced TNF secretion (**Fig. 2B**). Taking together, these data suggest that EGCG is capable of effectively inhibiting LPS-induced HMGB1 release in both macrophage and monocyte cultures.

To better understand EGCG's anti-inflammatory properties, we employed cytokine antibody array to examine its effects on LPS-induced release of multiple cytokines. At concentrations (15 µM) that completely abrogated LPS-induced HMGB1 release, EGCG did not affect LPS-induced release of G-CSF, IL-1α, P-selectin, or LIX in primary MuMACs (**Fig. 2C**). Consistent with few previous reports [32], [33], cytokine antibody array analysis revealed a dramatic suppression of LPS-induced release of TNF and IL-12 in primary MuMACs (**Fig. 2C**). Furthermore, EGCG dramatically inhibited LPS-induced release of IL-6, and a number of chemokines including MIP-1α, MIP-1γ, MIP-2, RANTES, KC, MCP1, and CXCL16 (**Fig. 2C**). Taken together, these experimental data suggest that EGCG selectively inhibits LPS-induced release of HMGB1, TNF, IL-6, IL-12, and chemokines, without affecting LPS-induced release of G-CSF, P-selection, LIX, IL-1α or nitric oxide.

### Delayed administration of EGCG still attenuated endotoxin-induced HMGB1 release

**Figure 3:**
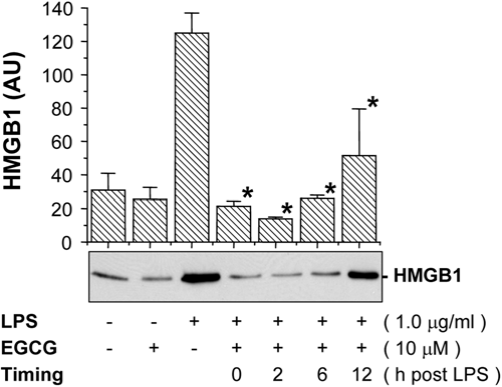
Delayed administration of EGCG still significantly attenuated endotoxin-induced HMGB1 release. Murine macrophage-like RAW 264.7 cells were stimulated with LPS, and EGCG (10 µM) was added at 0, 2, 6, and 12 hours post LPS stimulation. Levels of HMGB1 levels in the culture medium were determined at 16 hours after LPS stimulation, and expressed (in arbitrary unit, AU) as mean±S.D. of two independent experiments (N = 2). Shown in the lower panel was a representative Western blot. *, *P*<0.05 versus controls (“+ LPS alone”).

As compared with early proinflammatory cytokines (such as TNF), HMGB1 is released late following endotoxin stimulation [7]. It is intriguing to consider whether EGCG could inhibit HMGB1 release if added after LPS stimulation. Whereas concurrent administration of EGCG was most effective in inhibiting LPS-induced HMGB1 release, significant inhibition was still achieved when it was added 2 to 6 h after LPS (**Fig. 3**). It thus becomes feasible to attenuate late-acting proinflammatory mediators (such as HMGB1) by strategically administering EGCG in a delayed fashion.

### Determination of structure-function relationships

**Figure 4:**
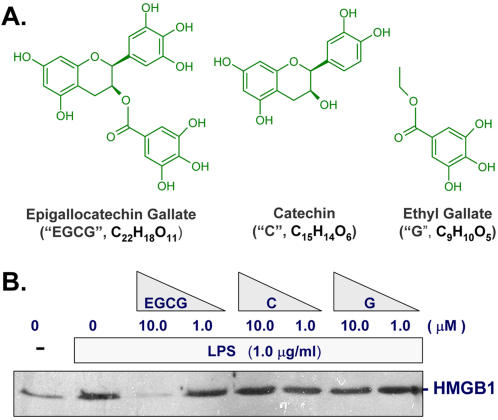
EGCG, but not catechin or ethyl gallate, effectively abrogated endotoxin-induced HMGB1 release. Macrophage cultures were stimulated with LPS in the absence, or presence of epigallocatechin gallate (EGCG), catechin (C), or ethyl gallate (G) (Panel A), and assayed for HMGB1 release by Western blotting analysis (Panel B) at 16 h post LPS stimulation. Note that epigallocatechin gallate, but not catechin or ethyl gallate, abrogated LPS-induced HMGB1 release.

As a class of biologically active polyphenols, catechins contain two or more aromatic rings (each carrying at least one aromatic hydroxyl) connected with a carbon bridge (consisting of five carbons and one oxygen, **Fig. 4A**). To gain insights into the structure-function relationships, we compared the HMGB1-inhibiting activities between EGCG and two relevant molecules: catechin and ethyl gallate (**Fig. 4A**). Even at concentrations up to 10 µM, catechin or ethyl gallate did not affect LPS-induced HMGB1 release (**Fig. 4B**), indicating that functional groups of both catechin and gallate are needed for EGCG's HMGB1-inhibiting properties.

### EGCG protected mice against lethal endotoxemia

**Figure 5:**
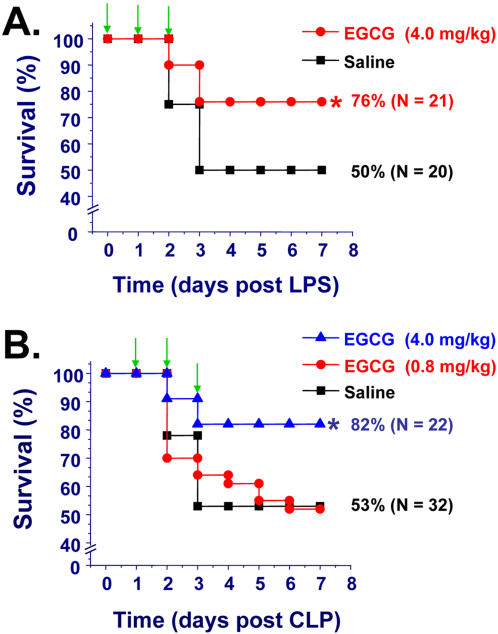
EGCG significantly protects mice against lethal endotoxemia (Panel A) and lethal sepsis (Panel B). Balb/C mice were subjected to lethal endotoxemia (LPS, 15 mg/kg, i.p., Panel A), or sepsis (induced by CLP, Panel B). At −0.5, +24, and +48 hours post the onset of endotoxemia, or +24, +48, +72 hours post the onset of sepsis, animals were intraperitoneally administered with saline (0.2 ml/mouse), or EGCG (0.2 ml/mouse, at indicated doses), and animal survival was monitored for up to two weeks. The Kaplan-Meier method was used to compare the differences in mortality rates between groups. *, *P*<0.05 versus saline.

In light of the capacity of EGCG in attenuating LPS-induced HMGB1 release, we explored its efficacy in animal model of lethal endotoxemia. By treating animals with three doses of EGCG at −0.5, +24, and +48 hours post intraperitoneal administration of L.D._50_ dose of LPS, we observed a significant improvement in animal survival rate (from 50% to 76%, *P*<0.05, **Fig. 5A**), confirming a previous observation that mixture of tea catechins improved survival rate at 24 hour post onset of endotoxemia [32], [33].

### EGCG rescues mice from lethal sepsis

Although endotoxemia is useful for investigating the complex cytokine cascades, more clinically relevant animal models are necessary to explore therapeutic agents for the treatment of human sepsis. One well-characterized, standardized animal model of sepsis is induced by CLP. In light of the late and prolonged kinetics of HMGB1 accumulation in experimental sepsis [11], we reasoned that it might be possible to rescue mice from lethal sepsis even if EGCG is administered after the onset of sepsis. The first dose of EGCG was given 24 h after the onset of sepsis, a time point at which mice developed clear signs of sepsis (including lethargy, diarrhea, and piloerection). Repeated administration of EGCG beginning twenty-four hours *after* the onset of sepsis (followed by additional doses at 48, and 72 hours post sepsis) conferred a dose-dependent protection against lethal sepsis (N = 22-32 mice per group, **Fig. 5B**), significantly increasing animal survival rate from 53% to 82% (*P*<0.05), supporting a therapeutic potential for EGCG in the treatment of sepsis.

### EGCG attenuates sepsis-induced systemic HMGB1 accumulation

**Figure 6:**
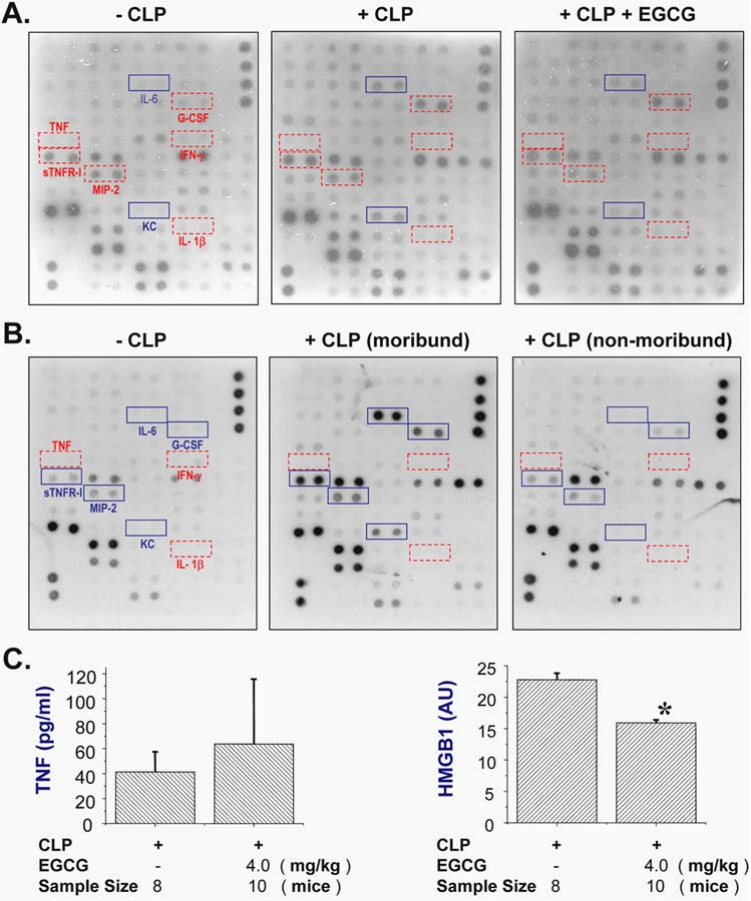
EGCG attenuates sepsis-induced systemic accumulation of IL-6, KC, and HMGB1. Balb/C mice were subjected to lethal sepsis by CLP, and intraperitoneally administered with control saline (0.2 ml/mouse) or EGCG (4.0 mg/kg) at +24, +48 hours post CLP. At 52 hours post the onset of sepsis, serum levels of cytokines (Panel A, B, C), or HMGB1 (Panel C) were determined by cytokine antibody array (Panel A, B), ELISA (Panel C), or Western blotting analysis (Panel C), respectively. In parallel experiments, serum were pooled from 3 normal mice (-CLP), 3 septic mice approaching moribund state (52 h post CLP), 3 septic mice in non-moribund state (52 h post CLP), and assayed for cytokine profile by antibody array (Panel B). Serum levels of HMGB1 or TNF (Panel C) were expressed as mean±SD (n = 8–10). *, *P*<0.05 (ANOVA, Tukey test).

To gain insight into its protective mechanism, we evaluated the effects of EGCG on the systemic accumulation of various cytokines by cytokine antibody array, ELISA, and Western blotting analysis. Delayed administration of EGCG did not affect the circulating levels of most cytokines (**Fig. 6A**), but significantly attenuated circulating levels of IL-6 (83.5±4.5% of positive controls, “CLP+Saline”; versus 20.0±11.0% of positive controls, “CLP+EGCG”; n = 2, *P*<0.01) and KC (26.5±13.5% of positive controls, “CLP+Saline”; versus 11.4±8.6% of positive controls, “CLP+EGCG”; n = 2, *P*<0.05). To evaluate the significance of IL-6 and KC in predicting outcome of lethal sepsis [34], we compared their circulating levels in dying (in a moribund state) versus non-dying (in a non-moribund state) septic mice at 52 h post CLP. Circulating levels of IL-6 and KC, as well as MIP-2, sTNFR-I and G-CSF, in dying septic mice (“+ CLP, moribund”, **Fig. 6B** **, middle panel**) were markedly higher than those in healthy mice (“-CLP”, **Fig. 6B** **, left Panel**), but dramatically lower than those in non-dying septic mice (“+ CLP, non-moribund”, **Fig. 6B** **, right panel**). It confirms the notion that plasma levels of IL-6, KC, MIP-2, and TNF soluble receptor I (sTNFR-I) were reliable predictors of lethal outcome in experimental [34], [35], or clinical sepsis [36].

Although delayed administration of EGCG did not attenuate circulating TNF levels at 52 h after the onset of sepsis (**Fig. 6C** **, left panel**), it did significantly attenuate circulating levels of HMGB1 (**Fig. 6C** **, right panel,** *P*<0.05), suggesting that EGCG confers protection against lethal sepsis partly by attenuating systemic HMGB1 accumulation.

### EGCG inhibits HMGB1-induced cytokine release

**Figure 7:**
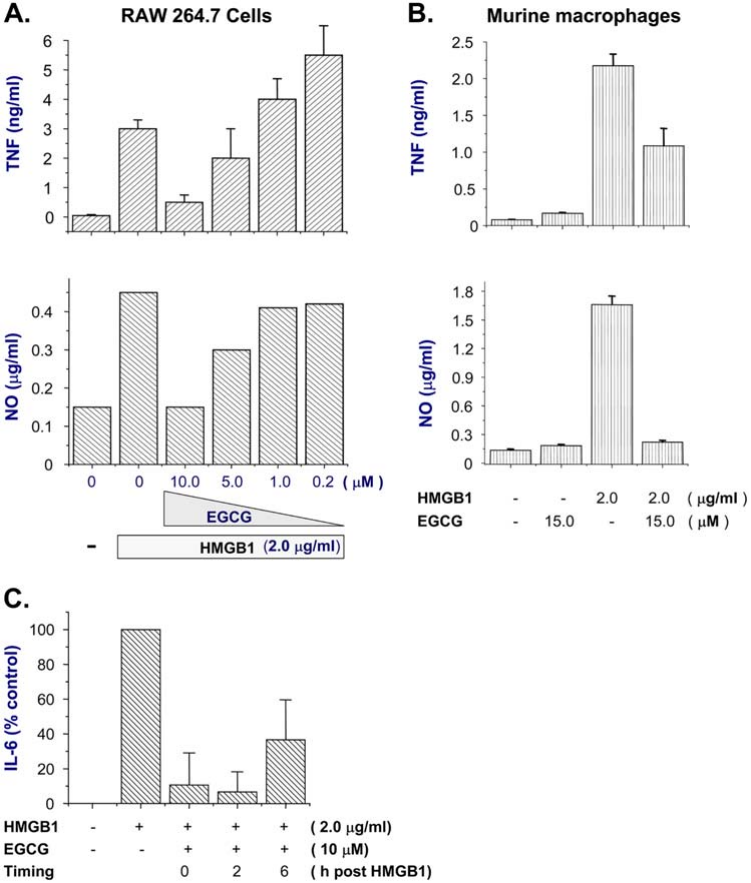
EGCG attenuates HMGB1-induced release of proinflammatory mediators. Murine macrophage-like RAW 264.7 cells (Panel A, Panel C) or primary murine peritoneal macrophages (Panel B) were stimulated with HMGB1 (2.0 µg/ml) in the absence, or presence of EGCG added at indicated concentrations, and time points post HMGB1 stimulation. At 16 hours after HMGB1 stimulation, levels of TNF (Panel A, B, top), IL-6 (Panel C), nitric oxide (Panel A, B, bottom) in the culture medium were determined by ELISA or Griess reaction (Panel A, B), respectively.

To elucidate additional mechanisms underlying EGCG-mediated protection, we determined whether EGCG inhibits HMGB1-mediated inflammatory response. Indeed, EGCG dose-dependently inhibited HMGB1-induced TNF release in murine macrophage-like RAW 264.7 cells (**Fig. 7A** **, top panel**). Despite the fact that EGCG failed to inhibit LPS-induced nitric oxide (**Fig. 1C**), it dose-dependently suppressed HMGB1-induced release of nitric oxide in RAW 264.7 cells (**Fig. 7A**, bottom panel), or primary MuMACs (**Fig. 7B**), supporting the notion that LPS and HMGB1 use distinct mechanisms to activate innate immune cells [29], [37]. Furthermore, EGCG effectively inhibited HMGB1-induced release of IL-6 release, even when it was given 2–4 hours after HMGB1 stimulation (**Fig. 7C**, and data not shown). Taken together, these data suggest that EGCG confers protection against lethal sepsis partly by inhibiting HMGB1 cytokine activities.

### EGCG prevents clustering of exogenous HMGB1 on macrophage cell surface

**Figure 8:**
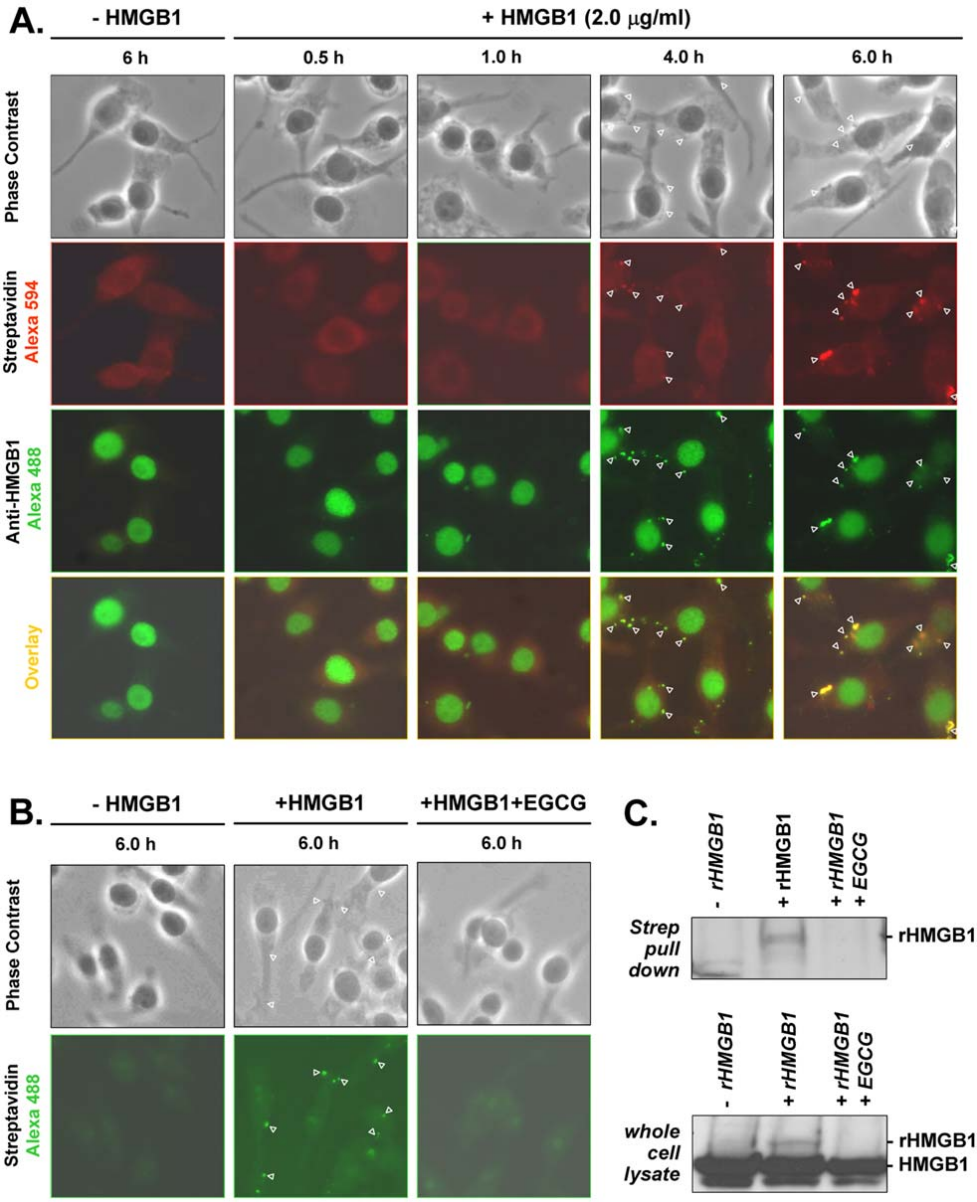
EGCG prevents HMGB1 accumulation/clustering on macrophage cell surface. Macrophage cultures were incubated with biotin-labeled CBP-HMGB1 fusion protein (“+ HMGB1”, 2 μg/ml), in the absence (Panel A), or presence (Panel B, C) of EGCG (10 µM) for various period of time. To visualize exogenous HMGB1, cells were stained with streptavidin-conjugated Alexa 594 (Panel A, “Streptavidin Alexa 594”) or Alexa 488 (Panel B, “Streptavidin Alexa 488” ), or HMGB1-specific rabbit antibodies plus Alexa 488-conjugated goat-anti-rabbit antibodies (Panel A, “Anti-HMGB1 Alexa 488”). Phase contras images indicate macrophage cell morphology; overlay images show co-localization of red and green fluorescence (as yellow). Note anti-HMGB1 antibody-specific immunostaining revealed the presence of both exogenous (on cell surface) and endogenous HMGB1 (in the nucleus) at 4–6 hours post HMGB1 incubation (Panel A, “Anti-HMGB1 Alexa 488”). To determine the relative content of exogenous HMGB1, streptvidin-pulled down fraction or whole cell lysate were immunoblotted with HMGB1-specific antibodies (Panel C). Note EGCG dramatically decreased levels of exogenous HMGB1 (indicated by the 33 kDa band corresponding to CBP-HMGB1 fusion protein) (“rHMGB1”).

Engagement of LPS to cell-surface receptor (such as CD14, TLR4) induces clustering of ligand/receptor complexes (consisting of TLR4, hsp70, hsp90, CXCR4, and GDF5) at the cell surface [38], which is critical for signaling transduction, as receptor clustering-disrupting agents (such as nystatin or MCD) prevent LPS-induced cytokine production [38]. To gain insight into the mechanism by which EGCG attenuates HMGB1-mediated cytokine production, we first determined whether exogenous HMGB1 accumulates and clusters on macrophage cell surface. Biotin-labeled recombinant HMGB1 was used to distinguish exogenous (CBP-HMGB1 fusion) from endogenous HMGB1 protein. In the absence of exogenous HMGB1, staining with streptavidin-conjugated Alexa 488 or Alexa594 revealed weak and diffuse background fluorescence throughout the cytoplasmic region (**Fig. 8A, 8B**, “- HMGB1”). At 2-6 h post HMGB1 treatment, staining with streptavidin-Alexa 594 showed strong, punctuate fluorescence predominantly on macrophage cell surface (**Fig. 8A**, “Streptavidin Alexa 594”). These Alexa 594-associated red fluorescence co-localized with green fluorescence produced with HMGB1-specific antibodies (**Fig. 8A**, “Anti-HMGB1 Alexa 488”, and “Overlay”), indicating that exogenous HMGB1 accumulates and clusters on macrophage cell surface in a time-dependent fashion. Intriguingly, this HMGB1-induced self clustering coincided with the kinetics of HMGB1-mediated cytokine release (e.g., TNF), which begins at 4-6 h, and peaks around 16 h post HMGB1 stimulation (data not shown).

To further elucidate the mechanism by which EGCG attenuates HMGB1-mediated cytokine production, we determined whether EGCG affects HMGB1-induced self accumulation/clustering on macrophage cell surface. Indeed, in the presence of EGCG (10 µM), HMGB1-induced cell surface clustering, as indicated by Alexa 488-associated cell surface fluorescence, was almost completely eliminated (**Fig. 8B**), suggesting that EGCG prevents HMGB1 accumulation on macrophage cell surface. To test this possibility, we assayed macrophage whole-cell lysate or streptavidin pull-down fraction for content of exogenous HMGB1 by Western blotting analysis. As expected, at 6 hours following HMGB1 incubation, levels of exogenous HMGB1 in macrophage whole-cell lysate or streptavidin-pulled-down fractions were increased in the presence of exogenous HMGB1 (“+rHMGB1”, **Fig. 8C**), but dramatically decreased in the presence of EGCG (“+rHMGB1+EGCG”, **Fig. 8C**).

## Discussion

We recently discovered that green tea, brewed from the leaves of the plant, *Camellia sinensis*, is effective in inhibiting bacterial endotoxin-induced HMGB1 release [31], but the active components responsible for its activities were previously unknown. Here we report that a major component, EGCG, recapitulated HMGB1-inhibiting activities of green tea, and dose-dependently inhibited LPS-induced HMGB1 release in macrophage/monocyte cultures. At concentrations that completely abrogated LPS-induced HMGB1 release, EGCG did not affect LPS-induced nitric oxide release, but only partially attenuated LPS-induced TNF secretion. These data contradict with some reports [39], [40], but agree with several other observations [32], [33]. The underlying causes for this discrepancy are unknown, and may be partly attributable to EGCG's chemical properties, which can spontaneously dimerize to liberate immunostimulatory products (such as hydrogen peroxide) [10], [41].

The mechanism underlying EGCG-mediated suppression of HMGB1 release remains elusive. For instance, it is not known whether EGCG-mediated suppression of HMGB1 release is dependent on its antioxidant activities, because some antioxidants (e.g., catechin, ethyl gallate) failed to inhibit LPS-induced HMGB1 release. Similarly, it is not yet known whether EGCG inhibits LPS-induced HMGB1 release through inhibiting LPS-induced cytoplasmic translocation, or post-translational modification (such as acetylation or phosphorylation). Interestingly, it has been suggested that EGCG can bind to lipid raft-associated cell surface receptor (e.g., the 67 kDa laminin receptor, 67LR) to confer its anti-cancer properties [42], [43], or anti-inflammatory allergic response [44], [45]. LPS can induce clustering of ligand/receptor complexes (containing hsp70, hsp90, CXCR4, GDF5 and TLR4) within membrane macrodomain (lipid rafts), which transmit signals to active macrophages to produce various proinflammatory mediators [38]. Since receptor clustering-disrupting agents (such as nystatin or MCD) can prevent LPS-induced cytokine production [38], it will thus be interesting to determine whether EGCG inhibits HMGB1 release via similar mechanisms.

Once released, extracellular HMGB1 employs several cell surface receptors (such as TLR2, TLR4, or RAGE) to activate innate immune cells to produce pro-inflammatory cytokines [46]–[49]. Indeed, fluorescence resonance energy transfer (FRET) analysis has demonstrated a close physical interaction between HMGB1 and TLR2 or TLR4 on macrophage cell surface within 5-15 minutes of HMGB1 incubation [47], long before subsequent HMGB1-induced cytokine release. Intriguingly, we observed a time-dependent accumulation of exogenous HMGB1 clustering on macrophage cell surface within 2–6 hours of HMGB1 incubation, which correlates with the kinetics of HMGB1-induced release of proinflammatory cytokines [29]. On the other hand, EGCG dose-dependently inhibited cell surface clustering of exogenous HMGB1, and consequently attenuated HMGB1-induced release of proinflammatory mediators (e.g., TNF, IL-6, and NO). Consistently, agents (e.g., catechin or ethyl gallate) incapable of inhibiting HMGB1-cell surface clustering uniformly failed to inhibit HMGB1-mediated cytokine production. We thus propose that HMGB1 may induce potential ligand/receptor (e.g., TLR2, TLR4, or RAGE) clustering on macrophage cell surface, which may be a prerequisite for HMGB1-mediated macrophage activation. Given the diverse range of receptors (e.g., TLR2, TLR4, or RAGE) involved in HMGB1 recognition [7], [50], [51], it is intriguing to investigate whether binding of HMGB1 to different receptors leads to combinational clustering of different receptors (such as TLR2 or TLR4) within the lipid rafts [38], [52], [53]. Nevertheless, our present study suggests a novel mechanism by which EGCG prevents HMGB1-mediated cytokine production–potentially by interfering with HMGB1-induced ligand/receptor clustering. Although EGCG-mediated suppression of HMGB1 cell surface clustering may not account for its inhibitory effects on LPS-induced HMGB1 release, it likely underlies its inhibitory effects on cytokine activities of the secreted HMGB1.

In light of the capacity of EGCG in inhibiting LPS-induced HMGB1 release and cytokine activities, we explored its efficacy in animal models of lethal endotoxemia and sepsis (induced by cecal ligation and puncture). Consistent with a previous observation that green tea polyphenols confer protection at 24 h post onset of endotoxemia [32], we found that EGCG promoted significant, and long-lasting protection against lethal endotoxemia. More importantly, delayed and repeated administration of EGCG, beginning at 24 h after onset of sepsis, significantly rescued mice from lethal sepsis, supporting a therapeutic potential of EGCG in the clinical management of human sepsis.

The pathogenesis of lethal sepsis remains obscure, but is mediated in part by excessive release of early (e.g., TNF and IL-1) and late (e.g., HMGB1) proinflammatory cytokines. Appearing relatively early in the circulation, TNF plays a protective role in sepsis [54], and its circulating levels do not correlate with lethality of experimental sepsis [34], [35], [55]. In contrast, dys-regulated inflammatory response sustained by late-acting mediators (such as HMGB1) may be more pathogenic in lethal sepsis. Because EGCG could inhibit LPS-induced TNF release *in vitro*, we strategically administered EGCG in a delayed fashion (at 24 h post CLP) to preserve a potentially beneficial early TNF response. Consequently, delayed administration of EGCG did not affect circulating levels of TNF at late stage of sepsis, but specifically attenuated systemic accumulation of HMGB1, as well as IL-6 and KC-two most reliable surrogate markers of lethal sepsis [34], [35]. In contrast to HMGB1, IL-6 and KC may not critically important in the pathogenesis of sepsis, because neither anti-IL-6 nor anti-KC antibodies confer long-lasting protection against lethal sepsis [56], [57]. Therefore, we propose that EGCG rescues mice from lethal sepsis partly through inhibiting systemic HMGB1 accumulation, as well as HMGB1-induced release IL-6 and KC.

In conclusion, we demonstrated a major tea component, EGCG, recapitulated green tea's HMGB1-inhibiting activities, and dose-dependently abrogated LPS-induced HMGB1 release in macrophage/monocyte cultures. Its beneficial effects in experimental sepsis were partly attributable to: 1) attenuation of systemic accumulation of proinflammatory mediator (e.g., HMGB1) and surrogate markers (e.g., IL-6 and KC) of lethal sepsis; and 2) suppression of HMGB1-mediated inflammatory responses by preventing accumulation of exogenous HMGB1 on macrophage cell surface. The doses of EGCG given to septic mice (4 mg/kg, i.e., 10 µM) are much higher than those readily available in humans (up to 1 µM) after ingestion of 1 cup of green tea ^58^. However, concentrated forms of de-caffeinated green tea extracts or purified EGCG are commercially available, and an individual does not need to drink multiple cups of tea everyday to enjoy the health benefits that green tea confers. It will be possible and important to evaluate the therapeutic potential of tea catechins (such as EGCG) for patients with lethal sepsis or other inflammatory diseases in future studies.
